# Frequency of genetic variants associated with arrhythmogenic right ventricular cardiomyopathy in the genome aggregation database

**DOI:** 10.1038/s41431-018-0169-4

**Published:** 2018-05-25

**Authors:** Charlotte L Hall, Henry Sutanto, Chrysoula Dalageorgou, William John McKenna, Petros Syrris, Marta Futema

**Affiliations:** Centre for Heart Muscle Disease, Institute of Cardiovascular Science, University College London, London, UK

## Abstract

Arrhythmogenic right ventricular cardiomyopathy (ARVC) is a rare inherited heart-muscle disorder, which is the most common cause of life-threatening arrhythmias and sudden cardiac death (SCD) in young adults and athletes. Early and accurate diagnosis can be crucial in effective ARVC management and prevention of SCD.

The genome Aggregation Database (gnomAD) population of 138,632 unrelated individuals was searched for previously identified ARVC variants, classified as pathogenic or unknown on the disease genetic variant database (http://www.arvcdatabase.info/), in five most-commonly mutated genes: *PKP2*, *DSP*, *DSG2*, *DSC2* and *JUP*, where variants account for 40–50% of all the ARVC cases. Minor allele frequency (MAF) of 0.001 was used to define variants as rare or common.

The gnomAD data contained 117/364 (32%) of the previously reported pathogenic and 152/266 (57%) of the unknown ARVC variants. The cross-ethnic analysis of MAF revealed that 11 previously classified pathogenic and 57 unknown variants were common (MAF ≥ 0.001) in at least one ethnic gnomAD population and therefore unlikely to be ARVC causing.

After applying our MAF analysis the overall frequency of pathogenic ARVC variants in gnomAD was one in 257 individuals, but a more stringent cut-off (MAF ≥ 0.0001) gave a frequency of one in 845, closer to the estimated phenotypic frequency of the disease.

Our study demonstrates that the analysis of large cross-ethnic population sequencing data can significantly improve disease variant interpretation. Higher than expected frequency of ARVC variants suggests that a proportion of ARVC-causing variants may be inaccurately classified, implying reduced penetrance of some variants, and/or a polygenic aetiology of ARVC.

## Introduction

Arrhythmogenic right ventricular cardiomyopathy (ARVC) is a heart muscle disorder that predominantly develops in the right ventricle, due to progressive replacement of myocardium by fatty or fibrofatty tissue leading to conduction disturbances and arrhythmias [1–3]. It is a rare disease with an estimated prevalence of 1 in 1000 to 1 in 5000 in the general population [4, 5], and accounts for about 11–22% of sudden cardiac death (SCD) cases among young athletes [6–8].

The variants detection rate in individuals who fulfil the Task Force diagnostic criteria [9] can reach 60% [10], with variants in desmosomal genes accounting for ~40–50% of cases [11]. The majority of the detected causal variants being located in the *PKP2* gene (~45–73% of all causal variants), which encodes Plakophilin-2 [12, 13]. Other commonly mutated genes in ARVC include *DSG2* (Desmoglein 2), *DSP* (Desmoplakin), *DSC2* (Desmocollin 2) and *JUP* (Junction Plakoglobin), which cause the disease mainly in the autosomal dominant mode, apart from the *JUP* gene and sometimes *DSP, DSC2* and more recently *DSG2* genes, where variants cause recessive ARVC [14–16].

Early diagnosis of ARVC based on clinical criteria remains challenging as SCD can present as either the first or final sign [17]. Genetic testing for ARVC variants offers the opportunity to identify individuals-at-risk in advance, before the clinical symptoms emerge. Next-generation sequencing (NGS) methods provide cost- and time-efficient genetic diagnosis, such as the recently developed genetic test for numerous inherited cardiovascular diseases, including ARVC [18]. However, in many cases difficulties in the interpretation of identified variants, mainly due to an insufficient evidence supporting their pathogenic effect, hinders accurate diagnosis. In 2015, the American College of Medical Genetics together with the Association of Molecular Pathology (ACMG–AMP) published guidelines to assist genetic diagnostic labs in evaluating the pathogenicity of variants [19]. The analysis of variant frequency in population data is the first ACMG–AMP criterion to be included in the variant interpretation process. Recent findings of the 1000 Genomes project based on a multi-ethnic whole-genome analysis strongly suggested that there is a bias in genetic studies of disease-associated variants [20], with the majority of variants being discovered in Caucasian populations. However, variants that appear to have a low frequency in Caucasians, which often supports their deleterious effect, can be common in other understudied ethnically diverse populations. Unless those have an advantageous effect, such as the sickle cell anaemia variants protecting from malaria infections [21], they are unlikely to be disease causing, due to negative selection. Although the efficacy of purifying selection may be affected by demographic history of a specific population, several studies demonstrated that this is less likely to have an effect on deleterious variants and that the selection is equally effective across human populations [22–24]. Therefore, examining rare allele frequencies across different ethnic groups can provide new insights into the pathogenicity status of some disease-associated variants. The importance of such analysis for correct disease diagnosis has been highlighted in a study of hypertrophic cardiomyopathy patients [25].

In 2013, Andreasen et al. attempted to examine the frequency of previously published ARVC variants using the NHLBI-Go Exome Sequencing Project (ESP, http://evs.gs.washington.edu/EVS/), which represents whole-exome sequencing data for 6500 individuals of two ethnic groups: European Americans and African Americans [26]. The observed genotype prevalence of ARVC variants in that report was one in five, which was at least 200-fold higher than the estimated phenotype prevalence of the disease. The authors suggested that such inflated ARVC variant frequency in the NHLBI-Go ESP cohort is likely to be due to incorrect classification of some previously published ARVC variants.

The frequency of *PKP2*, *DSG2*, *DSP* and *DSC2* variants was also evaluated, in 2016, in the Exome Aggregation Consortium (ExAC) data, a ten time larger whole-exome sequencing data set [27], however the variant classification was based on an in-house protocol, and variants of unknown significance were not examined.

In early 2017, genetic data from 123,136 individuals sequenced by whole-exome sequencing and 15,496 individuals sequenced by whole-genome sequencing was made available by the Genome Aggregation Database (gnomAD) investigators (http://gnomad.broadinstitute.org/about) [28]. To date, this is the largest and most comprehensive source of genetic information of individuals of different ethnic backgrounds to date.

The variant frequency analysis in separate ethnic groups as opposed to the global frequency in ExAC has been recently shown to be the most powerful approach for Mendelian disease variant frequency filtering and much more accurate than using the NHLBI-Go ESP cohort [28]. In this study we used the gnomAD population data set of 138,632 unrelated individuals, to investigate the frequency of previously reported ARVC variants in five of the most-commonly mutated ARVC genes, across eight different ethnic groups and in the gnomAD population as a whole. ARVC variants defined as pathogenic (*n* = 364) or unknown (*n* = 266) according to the manually curated ARVC genetic variants database maintained by the University Medical Center Groningen, the Netherlands [29], were analysed. Findings of this study should assist genetic diagnostic laboratories in the ARVC variant classification.

## Methods

### ARVC-associated variants

The ARVC/D Genetic Variants Database is a freely available collection of variants associated with ARVC and can be accessed via the link http://www.arvcdatabase.info/ [29]. The database’s variant classification is based on literature (clinical and experimental evidence) and in silico predictions. In this study variants classified as pathogenic and unknown, according to the ARVC genetic variants database curation and individually published classifications, which are not always supported by data from functional assays or family co-segregation, were extracted for the variant frequency analysis. Throughout this manuscript the term “pathogenic” was used as reported by the ARVC genetic variants database, which considers multiple published articles when classifying variants. The database was last updated in February 2015 and accessed in October 2017.

### gnomAD population data

The gnomAD database contains whole-exome sequencing data of 123,136 unrelated individuals sequenced by whole-exome sequencing and 15,496 unrelated individuals sequenced by whole-genome sequencing from several large-scale projects (http://gnomad.broadinstitute.org/about) [28]. It is the largest publically available population data set to date. Principal component analysis classified gnomAD participants to seven different ethnic groups: Non-Finnish European (EUR), Finnish (FIN), East Asian (EA), South Asian (SA), Latino (LAT), African (AFR) and Ashkenazi Jewish (AJ). A proportion (*N* = 3234) of the gnomAD population did not unambiguously cluster with any of the major populations and was classified as Other (OTH), which is likely to include individuals of mixed background.

### Data availability

The data sets generated and analysed during the current study are available in the Open Science Framework repository, https://osf.io/kg4br/#.

### Variant classification

The minor allele frequency (MAF) cut-off of 0.001, which is recommended for a dominant disease variant discovery in Mendelian diseases [30], was used to classify variants as rare (MAF < 0.001), frequency that supports variant’s pathogenic effect, and common (MAF ≥ 0.001), which are unlikely to cause ARVC. The cut-off allows for a higher than the estimated prevalence of ARVC in a general population (1 in 500 individuals, as opposed to 1 in 1000), however previous studies suggested that the disease penetrance can be significantly influenced by gender or environmental factors, such as exercise [31, 32], therefore the higher genotype frequency cut-off used in our study should allow for the possibility of reduced penetrance.

Based on the ARVC/D Genetic Variant Database data and the population frequency data from gnomAD, four classes of ARVC variants were distinguished: pathogenic rare (MAF < 0.001), pathogenic common (MAF ≥ 0.001), unknown rare (MAF < 0.001) and unknown common (MAF ≥ 0.001).

## Results

### Spectrum of ARVC variants in gnomAD

The ARVC/D Genetic Variants Database listed 364 pathogenic and 266 unknown variants across the five major ARVC genes: *PKP2*, *DSP*, *DSG2*, *DSC2* and *JUP*. The majority of different pathogenic variants were reported in the *PKP2* gene (*n* = 171) (summarised in Table 1).

**Table 1:** Summary of the ARVC pathogenic and unknown variants observed in the gnomAD data per gene.

Gene	Transcript ID	AAc	*N* of variants on the ARVC mutation database		Total *N* of variants in gnomAD	*N* of variants per AAc	*N* of ARVC-associated variants in gnomAD	
			Pathogenic	Unknown variants			Pathogenic (%)	Unknown variants (%)
*DSC2*	ENST00000280904.10—NM_024422	901	42	35	1374	1.53	19 (45)	20 (57)
*DSG2*	ENST00000261590.12—NM_001943	1118	50	60	1516	1.36	26 (52)	40 (67)
*DSP*	ENST00000379802.7—NM_004415	2871	86	96	3052	1.06	21 (24)	54 (56)
*JUP*	ENST00000393931.7—NM_002230	745	15	14	1127	1.51	10 (67)	8 (57)
*PKP2*	ENST00000070846.10—NM_004572	837	171	61	1338	1.6	41 (24)	30 (49)
TOTAL			364	266	8407		117 (32)	152 (57)

Of the previously reported 364 pathogenic variants 32% were present in gnomAD. The proportion of unknown ARVC variants was higher (49%)

*AAc* amino acid count

The mean coverage of the analysed genes on the gnomAD database was as follows: *DSP* = 83.5×, *DSG2* = 73.7×, *JUP* = 66.9×, *DSC2* = 73.0× and *PKP2* = 75.7×. Out of the 364 ARVC pathogenic variants 117 (32%) were identified in the gnomAD population, which was 83 variants more than in the previously analysed NHLBI-Go ESP cohort [26]. The number was much higher for the unknown variants where 152/266 (57%) of the variants were found in gnomAD. Previously reported pathogenic variants in *PKP2* and *DSP* were the least represented, *PKP2* 41/171 (24%) and *DSP* 21/86 (24%) of the variants found in the gnomAD population. In contrast, the majority 10/15 (67%) of the previously reported pathogenic *JUP* variants were present in the gnomAD population (Table 1). All ARVC-associated variants identified in gnomAD had passed the variant call quality threshold (PASS filter) as set by the gnomAD consortium [28].

The gnomAD database contained in total 8407 different variants (including common variants located in the untranslated regions, captured intronic and synonymous changes) located in the five ARVC genes, with *PKP2* having the highest number of variants per amino acid (1.60), and *DSP* being at the opposite end with 1.06 variants per amino acid (Table 1).

### Cross-ethnic ARVC variants frequency comparison

**Fig. 1:**
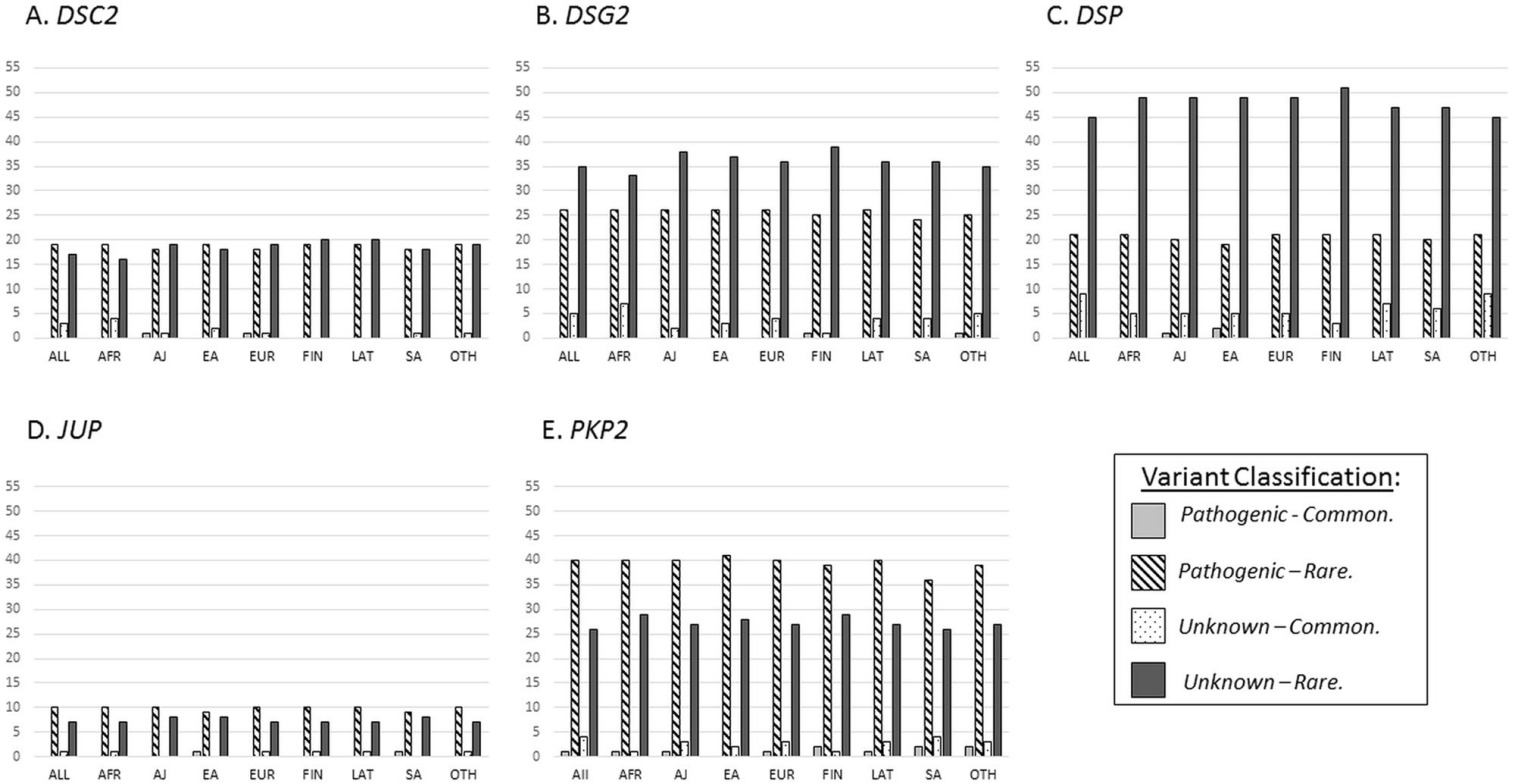
Distribution of the ARVC pathogenic and unknown variants classified as common (MAF ≥ 0.001) or rare (MAF < 0.001) by the gnomAD ethnic populations (AFR African, AJ Ashkenazi Jewish, EA East Asian;, EUR Non-Finnish European, FIN Finnish, LAT Latino, SA South Asian, OTH Other); and the gnomAD as a whole (indicated as ALL)

Frequencies of pathogenic and unknown variants were compared across seven ethnic groups (EUR, FIN, EA, SA, LAT, AFR and AJ) and the OTH group in gnomAD. The analysis showed that 11 out of 117 (9.4%) pathogenic variants located in the five ARVC genes occur at a higher frequency, i.e. are common (with MAF ≥ 0.001) in at least one ethnic group (Fig. 1 and Supplementary Table S1). However, only one pathogenic variant would be classified as common if the overall gnomAD frequency was used for the analysis. The highest proportion of these pathogenic common variants was found in *PKP2* (five variants) and *DSP* (three variants) genes.

The MAF ≥ 0.001 threshold classified 57 unknown variants as common, however 35 of them would still be rare if only the overall gnomAD frequency was taken into consideration. The percentage of unknown pathogenicity variants that were common in at least one of the analysed populations was higher in comparison to the pathogenic variants (34.2% vs. 9.4%). The highest proportion of unknown variants that could be classified as common, i.e. unlikely to have a causative effect, was found in *DSC2* followed by *DSP* (45% and 43%, respectively, of the identified unknown variants), whereas the lowest proportion of such variants was found in the *JUP* gene (one variant). Details of the unknown variants classified as common in at least one of the analysed ethnic populations are shown in Supplementary Table 2.

### Predictive analysis of common pathogenic variants

Variants previously classified as pathogenic that had MAF ≥ 0.001 in at least one of the gnomAD populations were further analysed using two in silico predictive algorithms (PolyPhen-2 [33], SIFT [34] and Clinvar database [35]). Only three of these variants, the *DSP* (c.2360 A > G) p.(Tyr787Cys), *PKP2* (c.176 A > T) p.(Gln59Leu) and *JUP* (c.1807G > T) p.(Val603Leu) had consistent prediction of being pathogenic according to PolyPhen-2 and SIFT, however, the ClinVar classification was either inconclusive or not available (Table S1). On the other hand, there were two variants in *PKP2:* (c.2431 C > A) p.(Arg811Ser), (c.1093 A > G) p.(Met365Val), one in *DSC2* (c.304 G > A) p.(Glu102Lys), and one in *DSP* (c.688 G > A p.(Asp230Asn) that were predicted as benign or tolerated by both in silico tools, and had conflicting interpretations as reported on ClinVar (Table S1).

There were 57 unknown common variants, of which 20 were predicted to be benign/tolerated by both PolyPhen-2 and SIFT. The variants are indicated in Supplementary Table S2.

### Overall frequency of ARVC variants

The overall ARVC genotype prevalence in the gnomAD population was established after excluding all unknown variants. Since there were no homozygous rare pathogenic variants found in the *JUP* gene, where variants are known to cause a recessive form of ARVC, and the possibility of identifying compound heterozygotes is not available in the gnomAD data set, *JUP* variants were also excluded (although there were 81 carriers of a rare pathogenic allele in the *JUP* gene, assuming that there were no compound heterozygotes). The pathogenic variants were classified as common or rare according to their frequency in the overall (global) gnomAD population and when each ethnic group was considered separately. Table 2 compares the number of alleles present in gnomAD between the two frequency analysis approaches.

**Table 2:** Comparison of ARVC allele counts (AC) of the pathogenic variants when using the overall gnomAD frequency (Global) and the cross-ethnic frequency (i.e. common/rare in at least one of the ethnic groups).

	Global		In at least one ethnic group	
	Common AC	Rare AC	Common AC	Rare AC
(A) MAF cut-off 0.001				
*DSC2*	0	374	207	167
*DSG2*	0	250	141	109
*DSP*	0	499	403	96
*PKP2*	631	523	987	167
TOTAL	631	1646	1738	539
(B) MAF cut-off 0.0001				
*DSC2*	325	49	336	38
*DSG2*	171	79	209	41
*DSP*	403	96	472	27
*PKP2*	965	189	1096	58
TOTAL	1864	413	2113	164

Two different MAF thresholds were used: (A) MAF ≥ 0.001 for common variants; (B) MAF ≥ 0.0001 for common variants. The overall prevalence of the ARVC genotype in 138,632 gnomAD individuals is 1 in 257, when using the MAF = 0.001 threshold, and 1 in 845 when the MAF = 0.0001 threshold was used. The reference sequences used were as follows: DSC2: ENST00000280904.10 (NM_024422), DSG2: ENST00000261590.12 (NM_001943), DSP: ENST00000379802.7 (NM_004415), JUP: ENST00000393931.7 (NM_002230) and PKP2: ENST00000070846.10 (NM_004572)

When applying the MAF cut-off of 0.001 to separate the rare from common variants, there are over three times more rare pathogenic alleles when using the global gnomAD frequency as opposed to looking at the ethnic groups separately (1646 vs. 539 variants) (Table 2A).

The number of rare pathogenic alleles across all the ethnic groups gave the overall prevalence of ARVC variants of one in 257 individuals (539 variants in 138,632 individuals). However, when applying a more rigorous MAF cut-off of 0.0001, which may be more appropriate for highly heterogeneous and penetrant diseases such as ARVC, as recently proposed by Whiffin et al. [36], the number of rare pathogenic alleles decreased to 164 (Table 2B). This gave the ARVC prevalence of one in 845 individuals, which is much closer to the observed 1 in 1000, and over 160 times lower than previously estimated in the smaller NHLBI-Go ESP cohort.

### New ARVC-associated variants

Since the ARVC genetic variants database was not updated after 2015, we reviewed recent literature (from 2015 until February 2018) using PubMed search terms ‘ARVC’, ‘mutation’ and ‘variant’. Eighty-nine variants that were not listed on the ARVC genetic variants database in the five ARVC genes were identified (Supplementary Table S3). Out of those novel variants 29 were found in the gnomAD population, however, none of the variants had MAF above the 0.001 cut-off in any of the gnomAD’s ethnic sub-groups. This suggests that researchers are becoming more aware of the importance of assessing variant frequency in large population data sets.

## Discussion

NGS has dramatically advanced the process of genetic variant identification; however, the clinical interpretation of identified variants remains a challenge. As more sequencing data are being generated this gives an opportunity to assess the frequency of a variant in a large and multi-ethnic cohort in order to assist the variant prediction process.

In this study, we used the largest available whole-exome/genome sequencing data set and analysed allele frequencies in each of the ethnic groups separately to review previously reported ARVC variants. This allowed us to assess the frequency of additional 83 (and 29 recently published, i.e. not included on the disease genetic variants database) ARVC-associated variants than in the previous study [26]. Pathogenic variants in *PKP2* and *DSP* genes were the least represented in gnomAD, which could suggest that variants in these genes have more deleterious effect in comparison to the remaining ARVC genes and are diluted by natural selection.

We demonstrated the importance of looking at the variant frequency per ethnic group as opposed to the overall gnomAD frequency. Such analysis pointed out 10 pathogenic variants that had MAF < 0.001 in the gnomAD population as a whole, but were common in at least one of the ethnic groups when analysed on group-by-group basis. Interestingly, one of the classified as common pathogenic variants, the *DSG2* (c.1003 A > G) p.(Thr335Ala), was recently identified in a homozygous form as a cause of recessive ARVC [15]. Furthermore, a more stringent MAF cut-off of 0.0001 (similar to the one recently suggested for hypertrophic cardiomyopathy [36]) would reclassify further 32 pathogenic variants as common.

We suggest that the status of variants previously classified as pathogenic that occur at a frequency higher than 0.001 in at least one of the gnomAD populations and that are predicted to be benign by in silico predictive algorithms is reviewed.

The analysis of the ARVC variant prevalence in gnomAD show that the ARVC-causing genotype was still higher than the observed disease frequency (one in 845 when MAF threshold of 0.0001 was used, or even higher, on in 257 when MAF was set to 0.001). This suggests that still a number of variants classified as pathogenic are not truly disease causing or are not fully penetrant.

There is also a possibility that some variants at a higher frequency may act as disease modifiers rather than causal variants, therefore may still be important for the phenotype interpretation. However, this requires further investigations involving deep-phenotyping and DNA-phenotype correlation analysis.

Our study summarises the evidence for variant interpretation process using three important criteria as listed by the ACMG–AMP guidelines: analysis of the variant frequency in the largest available population database, summary of gene-specific database for each variant (ClinVar) and results of the computational (in silico) predictions (PolyPhen2 and SIFT). These findings will be uploaded on to the ARVC genetic variants database (http://www.arvcdatabase.info/) with the next update.

### Limitations

ARVC has been reported across different ethnic populations, however, there is no evidence that its prevalence varies. Nevertheless, since environmental factors, which are likely to differ between some populations, can influence the ARVC phenotype expression, we cannot exclude the possibility that the disease prevalence also differs between some ethnic groups.

There is a chance that the ARVC genotype prevalence in gnomAD is slightly inflated since the analysis assume that each variant is found on a different allele (i.e. in separate individuals).

## Electronic supplementary material

Supplementary data
