# Supplementary material for: Frequency of genetic variants associated with arrhythmogenic right ventricular cardiomyopathy in the genome aggregation database
Source: Eur J Hum Genet. 2018 May 25;26(9):1312–8. doi: 10.1038/s41431-018-0169-4 (PMC6117313; doi:10.1038/s41431-018-0169-4)
Supplement: Supplementary file 1 — Supplementary data [file 41431_2018_169_MOESM1_ESM.docx]

**SUPPLEMENTARY DATA FOR:**

**Frequency of genetic variants associated with Arrhythmogenic Right Ventricular Cardiomyopathy (ARVC) in the genome Aggregation Database (gnomAD).**

Charlotte L Hall^1^**,** Henry Sutanto^1^**,** Chrysoula Dalageorgou**^1^,** William John McKenna**^1^**, Petros Syrris^1^, Marta Futema^1*^

^1^Centre for Heart Muscle Disease, Institute of Cardiovascular Science, University College London, London, UK

*Corresponding author

**Table S1. ARVC *pathogenic* variants with MAF ≥0.001 (*common*) in at least one gnomAD population.**

Two *in silico* mutation prediction tools (PolyPhen2 and SIFT) and ClinVar database were used to summarise the predicted effect of the genetic change. Ethnic populations abbreviated as follows: (AFR=African; AJ=Ashkenazi Jewish; EA=East Asian; EUR=Non-Finnish European; FIN=Finnish; LAT=Latino; SA=South Asian; OTH=Other;) and the gnomAD as a whole (indicated as Global). The reference sequences used were as follows: *DSC2*: ENST00000280904.10 (NM_024422), *DSG2*: ENST00000261590.12 (NM_001943), *DSP*: ENST00000379802.7 (NM_004415), *JUP*: ENST00000393931.7 (NM_002230), and *PKP2*: ENST00000070846.10 (NM_004572).

| **Gene** | **Variant** | **Population with MAF≥0.001** | **PolyPhen2** | **SIFT** | **ClinVar (number of submissions)** |
| --- | --- | --- | --- | --- | --- |
| *DSC2* | p.(Glu102Lys) (c.304G>A) | AJ, EUR | Benign | Tolerated | Conflicting interpretations of pathogenicity  Likely benign(3);Uncertain significance(4) |
| *DSG2* | p.(Thr335Ala) (c.1003A>G) | FIN, OTH | Possibly damaging | Tolerated | Conflicting interpretations of pathogenicity  Likely benign(1); Likely pathogenic(2); Uncertain significance(5) |
| *DSP* | p.(Tyr787Cys) (c.2360A>G) | EA | Probably damaging | Damaging | N/A |
| *DSP* | p.(Asp230Asn) (c.688G>A) | AJ | Benign | Tolerated | Conflicting interpretations of pathogenicity  Likely benign(1);Uncertain significance(3) |
| *DSP* | p.(Arg2639Gln) (c.7916G>A) | EA | Probably damaging | Tolerated | Conflicting interpretations of pathogenicity Benign(3);Likely benign(5);Uncertain significance(3) |
| *PKP2* | p.(Gln59Leu) (c.176A>T) | FIN | Probably damaging | Damaging | Uncertain significance (1) |
| *PKP2* | (c.1379-1G>A) | AFR | N/A | N/A | Conflicting interpretations of pathogenicity  Likely benign(1);Uncertain significance(1) |
| *PKP2* | p.(Met365Val) (c.1093A>G) | SA | Benign | Tolerated | Conflicting interpretations of pathogenicity  Likely benign(3);Uncertain significance(1) |
| *PKP2* | p.(Arg811Ser) (c.2431C>A) | AJ, OTH | Benign | Tolerated | Conflicting interpretations of pathogenicity Benign(1);Likely benign(1);Uncertain significance(5) |
| *PKP2* | p.(Ser140Phe)  (c.419C>T) | Global, FIN, EUR, LAT, OTH, SA | Benign | Damaging | Conflicting interpretations of pathogenicity  Likely benign(6);Uncertain significance(2) |
| *JUP* | p.(Val603Leu) (c.1807G>T) | EA, SA | Probably damaging | Damaging | Conflicting interpretations of pathogenicity  Likely benign(1);Uncertain significance(1) |

**Table S2. ARVC *unknown* variants with MAF ≥0.001 (*common*) in at least one gnomAD population.**

Two *in silico* mutation prediction tools (PolyPhen2 and SIFT) and ClinVar database were used to summarise the predicted effect of the genetic change. The variant predictions were: B=benign, PosD=possibly damaging, PD=probably damaging, T=tolerated, D=damaging. Ethnic populations abbreviated as follows: (AFR=African; AJ=Ashkenazi Jewish; EA=East Asian; EUR=Non-Finnish European; FIN=Finnish; LAT=Latino; SA=South Asian; OTH=Other;) and the gnomAD as a whole (indicated as Global). The reference sequences used were as follows: *DSC2*: ENST00000280904.10 (NM_024422), *DSG2*: ENST00000261590.12 (NM_001943), *DSP*: ENST00000379802.7 (NM_004415), *JUP*: ENST00000393931.7 (NM_002230), and *PKP2*: ENST00000070846.7 (NM_004572).

| **Gene** | **Protein change** | **Transcript change** | **Population with MAF≥0.001** | **PolyPhen2** | **SIFT** | **ClinVar (number of submissions)** |
| --- | --- | --- | --- | --- | --- | --- |
| *DSC2* | p.(Ala592Val) | (c.1775C>T) | AFR | B | T | Conflicting interpretations of pathogenicity  Likely benign(1);Uncertain significance(2) |
| *DSC2* | p.(Arg833His) | (c.2498G>A) | AFR | B | T | Conflicting interpretations of pathogenicity  Likely benign(1);Uncertain significance(3) |
| *DSC2* | p.(Phe58Val) | (c.172T>G) | AFR | B | T | Conflicting interpretations of pathogenicity  Likely benign(2);Uncertain significance(2) |
| *DSC2* | p.(Asp879Gly) | (c.2636A>G) | AFR | PD | D | Conflicting interpretations of pathogenicity  Likely benign(2);Uncertain significance(1) |
| *DSC2* | p.(Arg833Cys) | (c.2497C>T) | EA | PD | D | Conflicting interpretations of pathogenicity  Benign(2);Likely benign(1);Uncertain significance(1) |
| *DSC2* | p.(Gln638His) | (c.1914G>C) | AJ | B | D | Conflicting interpretations of pathogenicity  Likely benign(2);Uncertain significance(2) |
| *DSC2* | p.(Val303Met) | (c.907G>A) | Global, OTH, SA | PD | T | Conflicting interpretations of pathogenicity  Benign(1);Uncertain significance(2) |
| *DSC2* | p.(Leu732Val) | (c.2194T>G) | Global, EUR | B | T | Conflicting interpretations of pathogenicity  Benign(1);Likely benign(5);Uncertain significance(2) |
| *DSC2* | p.(Gly790del) | (c.2368_2370delGGA) | Global, EA | N/A | N/A | Conflicting interpretations of pathogenicity  Benign(3);Likely pathogenic(2);Uncertain significance(1) |
| *DSG2* | p.(Ala917Val) | (c.2750C>T) | SA | B | T | Likely benign (1) |
| *DSG2* | p.(Ala616Val) | (c.1847C>T) | EA | PD | T | Conflicting interpretations of pathogenicity  Likely benign(1);Uncertain significance(1) |
| *DSG2* | p.(Pro927Leu) | (c.2780C>T) | EA | PD | D | Uncertain significance (1) |
| *DSG2* | p.(Leu594Pro) | (c.1781T>C) | AJ | PosD | T | Conflicting interpretations of pathogenicity  Likely benign(2);Uncertain significance(1) |
| *DSG2* | p.(Asp435Asn) | (c.1303G>A) | SA | PD | T | Conflicting interpretations of pathogenicity  Likely benign(1);Uncertain significance(3) |
| *DSG2* | p.(Thr1070Met) | (c.3209C>T) | Global, EA, LAT, OTH | B | T | Conflicting interpretations of pathogenicity  Benign(1);Likely benign(2);Uncertain significance(2) |
| *DSG2* | p.(Val56Met) | (c.166G>A) | Global, EUR, LAT, OTH | PD | T | Conflicting interpretations of pathogenicity, risk factor  Likely benign(4);Pathogenic(1);Uncertain significance(4) |
| *DSG2* | p.(Val392Ile) | (c.1174G>A) | Global, EUR, OTH, SA | B | T | Conflicting interpretations of pathogenicity  Benign(1);Likely benign(4);Uncertain significance(1) |
| *DSG2* | p.(Val920Gly) | (c.2759T>G) | Global, EUR, LAT, OTH | PosD | T | Conflicting interpretations of pathogenicity  Benign(6);Likely benign(2);Uncertain significance(3) |
| *DSG2* | p.(Val158Gly) | (c.473T>G) | Global, FIN, EUR, LAT, OTH | B | D | Benign/Likely benign (9) |
| *DSG2* | p.(Gln731Pro) | (c.2192A>C) | AFR | B | T | Uncertain significance (3) |
| *DSG2* | p.(Val239Ala) | (c.716T>C) | AFR | PD | D | Uncertain significance (3) |
| *DSG2* | p.(His74Arg) | (c.221A>G) | AFR | PosD | T | Conflicting interpretations of pathogenicity  Likely benign(2);Uncertain significance(1) |
| *DSG2* | p.(Thr1099Ala) | (c.3295A>G) | AFR | B | T | Conflicting interpretations of pathogenicity  Benign(1);Likely benign(2);Uncertain significance(2) |
| *DSG2* | p.(Ser351Gly) | (c.1051A>G) | AFR | B | T | Benign/Likely benign (4) |
| *DSP* | p.(Ser2821Leu) | (c.8462C>T) | AJ | PosD | D | Uncertain significance (1) |
| *DSP* | p.(Asn956Tyr) | (c.2866A>T) | EA | B | D | Uncertain significance (2) |
| *DSP* | p.(Val2107Leu) | (c.6319G>C) | AFR | B | T | Uncertain significance (2) |
| *DSP* | p.(Tyr895His) | (c.2683T>C) | AFR | PD | T | Uncertain significance (2) |
| *DSP* | p.(Gln1648Arg) | (c.4943A>G) | EA | PosD | T | Likely benign (1) |
| *DSP* | p.(Arg808Cys) | (c.2422C>T) | AJ | PosD | T | Conflicting interpretations of pathogenicity  Likely pathogenic(1);Uncertain significance(2) |
| *DSP* | p.(Thr2267Ser) | (c.6799A>T) | EA | B | T | Conflicting interpretations of pathogenicity  Likely benign(1);Uncertain significance(1) |
| *DSP* | p.(Thr1217Met) | (c.3650C>T) | SA | PD | T | Conflicting interpretations of pathogenicity  Likely benign(4);Uncertain significance(1) |
| *DSP* | p.(Asn1865Tyr) | (c.5593A>T) | SA | PD | D | Conflicting interpretations of pathogenicity  Likely benign(5);Uncertain significance(1) |
| *DSP* | p.(Arg1497Trp) | (c.4489C>T) | AFR | PD | T | Conflicting interpretations of pathogenicity  Benign(1);Likely benign(3);Uncertain significance(1) |
| *DSP* | p.(Val2219Ile) | (c.6655G>A) | SA | B | T | Conflicting interpretations of pathogenicity  Likely benign(1);Uncertain significance(1) |
| *DSP* | p.(Gln90Arg) | (c.269A>G) | EA | PD | D | Conflicting interpretations of pathogenicity  Benign(1);Likely benign(4);Uncertain significance(3) |
| *DSP* | p.(Asn1726Lys) | (c.5178C>A) | SA | PosD | T | Conflicting interpretations of pathogenicity  Likely benign(8);Uncertain significance(1) |
| *DSP* | p.(Ala2294Gly) | (c.6881C>G) | LAT, OTH | PD | T | Conflicting interpretations of pathogenicity  Likely benign(6);Uncertain significance(4) |
| *DSP* | p.(Arg908His) | (c.2723G>A) | Global, AJ, EUR, LAT, OTH | PD | D | Conflicting interpretations of pathogenicity  Likely benign(7);Uncertain significance(2) |
| *DSP* | p.(Glu1740Lys) | (c.5218G>A) | Global, AJ, EUR, LAT, OTH | PD | T | Conflicting interpretations of pathogenicity  Likely benign(8);Uncertain significance(2) |
| *DSP* | p.(Val30Met) | (c.88G>A) | Global, EUR, OTH, SA | B | T | Conflicting interpretations of pathogenicity  Benign(1);Likely benign(5);Likely pathogenic(1);Pathogenic(1);Uncertain significance(6) |
| *DSP* | p.(Arg1458Gly) | (c.4372C>G) | Global, FIN, EUR, LAT, OTH | PosD | T | Conflicting interpretations of pathogenicity  Likely benign(7);Uncertain significance(5) |
| *DSP* | p.(Val1530Phe) | (c.4588G>T) | Global, FIN, OTH | B | D | Benign/Likely benign (6) |
| *DSP* | p.(Met2819Leu) | (c.8455A>C) | Global, EA, LAT, OTH | B | T | Conflicting interpretations of pathogenicity  Benign(1);Likely benign(5);Uncertain significance(2) |
| *DSP* | p.(Tyr2731His) | (c.8191T>C) | Global, SA | PD | T | Conflicting interpretations of pathogenicity  Benign(1);Likely benign(6);Uncertain significance(1) |
| *DSP* | p.(Gly939Ser) | (c.2815G>A) | Global, AFR, LAT, OTHR | B | T | Benign/Likely benign (12) |
| *DSP* | p.(Glu1833Val) | (c.5498A>T) | Global, AFR, AJ, FIN, EUR, LAT, OTH | PD | T | Conflicting interpretations of pathogenicity  Benign(7);Likely benign(4);Uncertain significance(1) |
| *JUP* | p.(Val648Ile) | (c.1942G>A) | Global, AFR, FIN, EUR, LAT, OTH | PosD | T | Benign (6) |
| *PKP2* | p.(Thr242Met) | (c.725C>T) | EA | B | T | Uncertain significance (2) |
| *PKP2* | p.(His877Gln) | (c.2631C>A) | SA | PD | D | Uncertain significance (2) |
| *PKP2* | p.(Ala474Val) | (c.1421C>T) | EA | B | T | Conflicting interpretations of pathogenicity  Likely benign(1);Uncertain significance(1) |
| *PKP2* | p.(Ala546Thr) | (c.1636G>A) | LAT | PosD | T | Conflicting interpretations of pathogenicity  Likely benign(1);Uncertain significance(2) |
| *PKP2* | p.(Asp829Asn) | (c.2485G>A) | AFR | PD | T | Benign/Likely benign (4) |
| *PKP2* | p.(Ser169Gly) | (c.505A>G) | Global, EUR, SA | B | T | Conflicting interpretations of pathogenicity  Likely benign(4);Uncertain significance(3) |
| *PKP2* | p.(Thr338Ala) | (c.1012A>G) | Global, AJ, OTH, SA | B | T | Conflicting interpretations of pathogenicity  Benign(3);Likely benign(2);Uncertain significance(2) |
| *PKP2* | p.(Val587Ile) | (c.1759G>A) | Global, AJ, EUR, LAT, OTH | PD | T | Conflicting interpretations of pathogenicity  Benign(2);Likely benign(5);Uncertain significance(1) |
| *PKP2* | p.(Glu58Asp) | (c.174G>T) | Global, AJ, FIN, EUR, LAT, OTH, SA | PosD | T | Benign/Likely benign (8) |

1. 0.25 0.50 0.75 1.00

**False Positive Rate (FPR) value**

Benign (B) 🡪 Possibly damaging (PosD) 🡪 Probably damaging (PD)

**PolyPhen2**

**Table S3. Recently published ARVC variants.**

PubMed database was search for publications published between 2015 and February 2018 using terms ‘ARVC’, ‘variant’, and ‘mutation’. The frequency of ARVC-associated variants that were not previously included on the ARVC mutation database were analysed using the gnomAD data. *In silico* mutation prediction tools (PolyPhen2 and SIFT) and ClinVar database were used to summarise the predicted effect of the genetic change. N/A means not available, i.e. not present on gnomAD. The variant predictions were: B=benign, PosD=possibly damaging, PD=probably damaging, T=tolerated, D=damaging. “-“ indicates prediction not possible for the type of variant. Ethnic populations abbreviated as follows: (AFR=African; AJ=Ashkenazi Jewish; EA=East Asian; EUR=Non-Finnish European; FIN=Finnish; LAT=Latino; SA=South Asian; OTH=Other;).

▲indicates published variants with incomplete information or incorrect nomenclature

| **Gene** | **Variant** | **Highest observed MAF** | **Population with highest MAF** | **PolyPhen2** | **SIFT** | **ClinVar (number of submissions)** |
| --- | --- | --- | --- | --- | --- | --- |
| *PKP2* | p.(Gly23Glu)  (c.68G>A) | N/A | N/A | B | T | Uncertain significance (1) |
| *PKP2* | p.(Val72Glyfs*40 )  (c.215del) | N/A | N/A | - | - | - |
| *PKP2* | p.(Pro105Leufs*7)  (c.314del) | N/A | N/A | - | - | - |
| *PKP2* | p.(Tyr119*)  (c.356dup) | N/A | N/A | - | - | - |
| *PKP2* | p.(Gly314Glu  (c.941G>A) | 0.00011082 | EUR | B | T | Uncertain significance (1) |
| *PKP2* | p.(Gln323Argfs*12)  (c.968_975delinsGCCTTT) | N/A | N/A | - | - | - |
| *PKP2* | p.(Ser368Ilefs*19)  (c.1101dup) | N/A | N/A | - | - | - |
| *PKP2* | p.(Ala372Thr)  (c.1114G>A) | 2.3682x10^-5^ | EUR | B | T | - |
| *PKP2* | p.(Phe376Alafs*8)  (c.1125_1132del) | N/A | N/A | - | - | Pathogenic (2) |
| *PKP2* | p.(Val391Thrfs*6)  (c.1171_1378del) | N/A | N/A | - | - | - |
| *PKP2* | p.(Asn427Ilefs*7)  (c.1255_1279dup) | N/A | N/A | - | - | - |
| *PKP2* | p.(Ile458Glnfs*7)  (c.1372_1375del) | N/A | N/A | - | - | - |
| *PKP2* | p.(Val587Thrfs*72)  (c.1748_1755dup) | N/A | N/A | - | - | - |
| *PKP2* | p.(Ser585*)  (c.1754C>G) | N/A | N/A | - | - | - |
| *PKP2* | p.(Tyr631Phefs*26)  (c.1892delinsTCC) | N/A | N/A | - | - | - |
| *PKP2* | p.(Asn634Thrfs*22)  (c.1901del) | N/A | N/A | - | - | - |
| *PKP2* | p.(Gly646*)  (c.1917_1935dup) | N/A | N/A | - | - | - |
| *PKP2* | p.(Glu657Serfs*27)  (c.1968del) | N/A | N/A | - | - | - |
| *PKP2* | p.(Glu667*)  (c.1999G>T) | 8.9607x10^-6^ | EUR | - | - | Pathogenic(1) |
| *PKP2* | p.(Tyr686*)  (c.2058T>A) | N/A | N/A | - | - | - |
| *PKP2* | p.(Tyr831*)  (c.2493T>A) | N/A | N/A | - | - | - |
| *PKP2* | p.(Gln173*)  (c.517C>T) | N/A | N/A | - | - | - |
| *PKP2* | p.(Gly5fs)  (c.14delG) | N/A | N/A | - | - | Pathogenic/Likely Pathogenic (2) |
| *PKP2* | p.(Gln617*)  (c.1828A>G) | N/A | N/A | - | - | Uncertain significance (1) |
| *PKP2* | p.(Ser151fs)  (c.451delT) | N/A | N/A | - | - | - |
| *PKP2* | p.(Ile681fs)  (c.2043delT)▲ | N/A | N/A | - | - | - |
| *PKP2* | p.(Val321Glyfs*11) (c.962_972delTCGGCCAGGCG)▲ | N/A | N/A | - | - | - |
| *PKP2* | (c.2194C>T)▲ | N/A | N/A | - | - | - |
| *PKP2* | (c. 810_813delGGTC)▲ | N/A | N/A | - | - | - |
| *JUP* | p.(Arg84Gln)  (c.251G>A) | 0.00010468 | SA | PD | D | - |
| *JUP* | p.Ser99Leu  (c.296C>T) | 0.00019701 | OTH | B | T | - |
| *JUP* | p.(Glu138Lys)  (c.412G>A) | 8.4955X10^-5^ | AFR | PD | D | - |
| *JUP* | p.Arg177Trp  (c.529C>T) | 0.000127621 | EUR | PD | D | Uncertain Significance (1) |
| *JUP* | p.(Arg177Gln)  (c.530G>A) | 6.41684X10^-5^ | EUR | B | T | Uncertain Significance (1) |
| *JUP* | p.(Arg526His)  (c.1577G>A) | 0.00013234 | SA | B | T | - |
| *JUP* | p.(Arg572Gln)  (c.1715G>A) | 0.00010599 | EA | PD | D | - |
| *JUP* | p.(Glu639Lys)  c.1915G>A | 4.2042x10^-5^ | AFR | PD | T | - |
| *DSC2* | p.(Ser67Gly)  (c.199A>G) | N/A | N/A | B | D | - |
| *DSC2* | p.(Glu207Gln)  (c.619G>C) | 2.69x10^-5^ | EUR | B | T | - |
| *DSC2* | p.(Glu210Asp)  (c.630G>C) | N/A | N/A | B | T | - |
| *DSC2* | p.(Val384Leu)  (c.1150G>C) | N/A | N/A | B | T | - |
| *DSC2* | p.(Met589Thr)  (c.1766T>C) | 0.000155 | OTH | B | T | Uncertain Significance (3) |
| *DSC2* | p.(Tyr646*)  (c.1938T>G) | 1.59x10^-5^ | EUR | - | - | Likely Pathogenic (1) |
| *DSC2* | p.(Gly799Glu)  (c.2396G>A) | N/A | N/A | B | D | Uncertain Significance (1) |
| *DSC2* | p.(Gly863fs)  (c.2582_2585dupGAAG) | 2.69x10^-5^ | EUR | - | - | - |
| *DSC2* | (c. 1445G>C)▲ | N/A | N/A | - | - | - |
| *DSG2* | p.(Arg49Cys)  (c.145C>T) | 3.2487x10^-5^ | SA | PD | D | Likely Pathogenic (1) |
| *DSG2* | p.(Glu60Lys)  (c.178G>A) | N/A | N/A | PD | D | Uncertain significance (1) |
| *DSG2* | p.(Gly166Trpfs*4)  (c.495dup) | 8.3738x10^-5^ | AFR | - | - | - |
| *DSG2* | (c.523+1G>A) | N/A | N/A | - | - | Likely Pathogenic (1) |
| *DSG2* | p.(Asn284Lysfs*4)  (c.852_855del) | N/A | N/A | - | - | - |
| *DSG2* | p.(Ser303Phe)  (c.908C>T) | 1.7925x10^-5^ | EUR | PD | D | Uncertain significance (1) |
| *DSG2* | p.(Thr589Lysfs*31)  (c.1765_1766insAA) | N/A | N/A | - | - | - |
| *DSG2* | p.(Asp778Tyr)  (c.2332G>T) | N/A | N/A | PD | D | - |
| *DSG2* | p.(Gln1114His)  (c.3342G>T) | N/A | N/A | B | T | - |
| *DSG2* | p.(Glu156fs)  (c.464_465insT) | 2.978x10^-5^ | LAT | - | - | Pathogenic (1) |
| *DSG2* | (c.523+1G>A) | N/A | N/A | - | - | Likely pathogenic (1) |
| *DSG2* | (c.1423+2T>G) | N/A | N/A | - | - | - |
| *DSP* | p.(Gln139*)  (c.415C>T) | N/A | N/A | - | - | - |
| *DSP* | p.(Arg315Pro)  (c.944G>C) | N/A | N/A | PD | D | - |
| *DSP* | p.(Gln357Alafs*13)  (c.1068dup) | N/A | N/A | - | - | - |
| *DSP* | p.(Ile368Thr)  (c.1103T>C) | 7.902x10^-5^ | EUR | PD | T | Uncertain Significance (1) |
| *DSP* | p.(Asn375Lysfs*9)  (c.1124dup) | N/A | N/A | - | - | - |
| *DSP* | p.(Ile399Argfs*44)  (c.1188_1195dup) | N/A | N/A | - | - | - |
| *DSP* | p.(Arg451His)  (c.1352G>A) | N/A | N/A | PD | D | Uncertain Significance (2) |
| *DSP* | p.(Cys482Tyr)  (c.1445G>A) | 8.9545x10^-6^ | EUR | PD | T | - |
| *DSP* | p.(Tyr587Asn)  (c.1759T>A) | N/A | N/A | PD | T | - |
| *DSP* | P(.Cys682*)  (c.2046C>A) | N/A | N/A | - | - | - |
| *DSP* | p.(Glu721Lys)  (c.2161G>A) | 8.9633x10^-6^ | EUR | B | T | - |
| *DSP* | p.(Ile870Thr)  (c.2609T>C) | 6.5342x10^-5^ | AFR | PosD | D | - |
| *DSP* | p.(Leu933Phe)  (c.2799G>C) | 0.0001581 | EUR | PD | D | Uncertain Significance (1) |
| *DSP* | p.(Arg1045*)  (c.3133C>T) | N/A | N/A | - | - | Pathogenic (1) |
| *DSP* | p.(Tyr1065*)  (c.3195C>G) | N/A | N/A | - | - | Pathogenic (1) |
| *DSP* | p.(Lys1110Argfs*5)  (c.3329del) | N/A | N/A | - | - | - |
| *DSP* | p.(Asp1248Lysfs*7)  (c.3735_3741dup) | N/A | N/A | - | - | Pathogenic (2) |
| *DSP* | p.(Glu1493*)  (c.4477G>T) | N/A | N/A | - | - | - |
| *DSP* | P(.Glu1501*)  (c.4501G>T) | N/A | N/A | - | - | - |
| *DSP* | p.(Ser1623Cys)  (c.4868C>G) | N/A | N/A | PD | D | - |
| *DSP* | p.(Arg1666Trp)  (c.4996C>T) | 0.00019399 | FIN | PD | D | - |
| *DSP* | p.(Lys1887Glufs*2)  (c.5659_5660del) | N/A | N/A | - | - | - |
| *DSP* | p.(Ile2040Alafs*18)  (c.6118_6121del) | N/A | N/A | - | - | - |
| *DSP* | p.(Pro2061Ser)  (c.6181C>T) | N/A | N/A | PD | T | - |
| *DSP* | p.(Gly2338Arg)  (c.7012G>A) | N/A | N/A | PD | D | - |
| *DSP* | p.(Gly2647Asp)  (c.7940G>A) | 8.9802x10^-6^ | EUR | PD | D | - |
| *DSP* | p.(Lys2693Profs*3)  (c.8077_8080del) | N/A | N/A | - | - | - |
| *DSP* | p.(Met2707Thr)  (c.8120T>C) | 3.2487x10^-5^ | SA | B | T | - |
| *DSP* | p.(Tyr2770Cys)  (c.8309A>G) | 5.3723x10^-5^ | EUR | PD | T | - |
| *DSP* | p.(Ile2797Thr)  (c.8390T>C) | 0.00010599 | EA | B | T | - |
| *DSP* | p.(Arg1738*)  (c.5212C>T) | N/A | N/A | - | - | - |

1. 0.25 0.50 0.75 1.00

**False Positive Rate (FPR) value**

Benign (B) 🡪 Possibly damaging (PosD) 🡪 Probably damaging (PD)

**PolyPhen2**
